# Supplementary material for: The causal role of circulating inflammatory markers in osteoporosis: a bidirectional Mendelian randomized study
Source: Front Immunol. 2024 Jul 18;15:1412298. doi: 10.3389/fimmu.2024.1412298 (PMC11291241; doi:10.3389/fimmu.2024.1412298)
Supplement: Supplementary file 1 [file DataSheet_1.zip › supplementary material/OSTEOPOROSIS-CIM/GCST90274774.tsv.gz_forest.pdf]

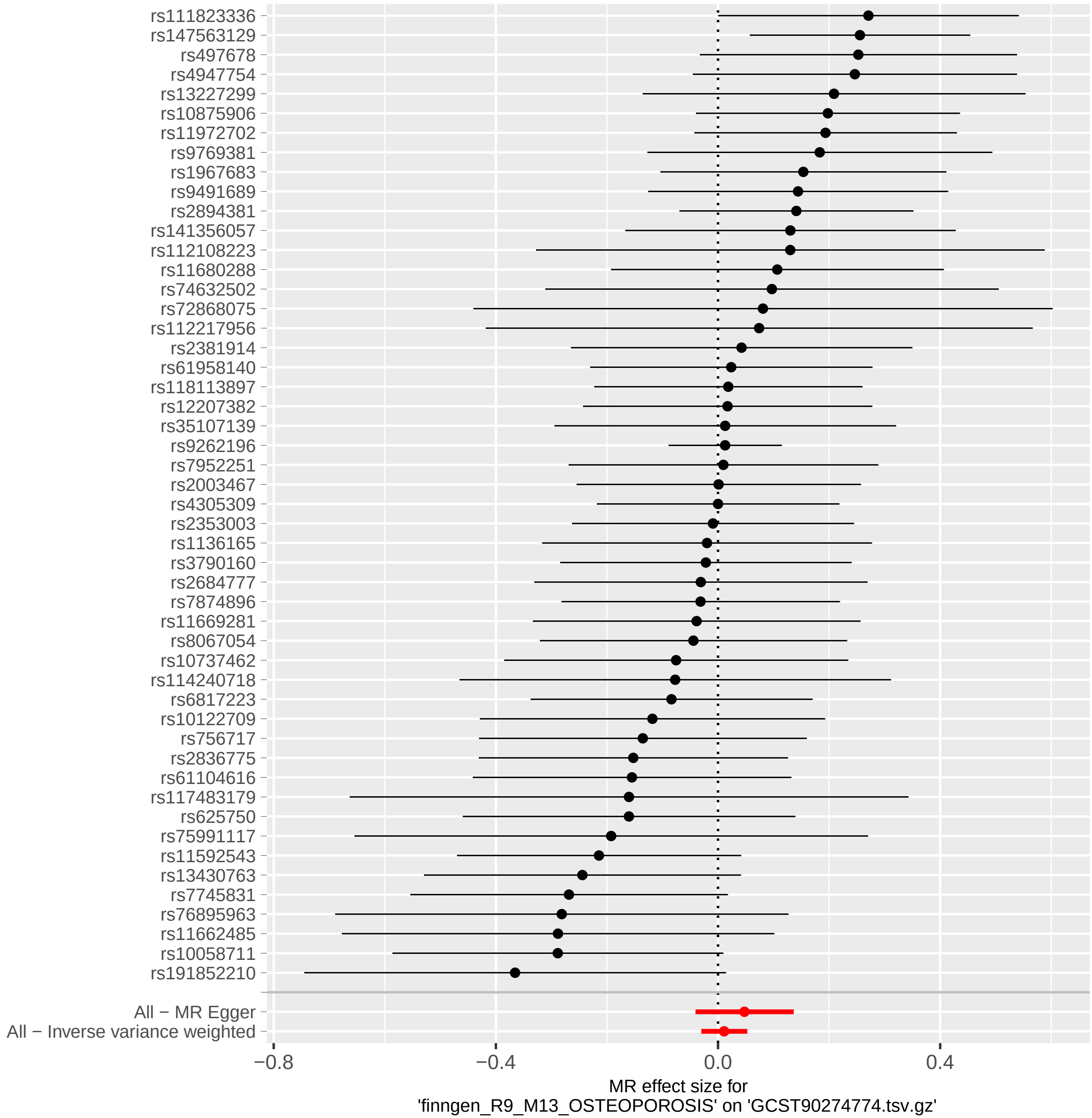
rs111823336
rs147563129
rs497678
rs4947754
rs13227299
rs10875906
rs11972702
rs9769381
rs1967683
rs9491689
rs2894381
rs141356057
rs112108223
rs11680288
rs74632502
rs72868075
rs112217956
rs2381914
rs61958140
rs118113897
rs12207382
rs35107139
rs9262196
rs7952251
rs2003467
rs4305309
rs2353003
rs1136165
rs3790160
rs2684777
rs7874896
rs11669281
rs8067054
rs10737462
rs114240718
rs6817223
rs10122709
rs756717
rs2836775
rs61104616
rs117483179
rs625750
rs75991117
rs11592543
rs13430763
rs7745831
rs76895963
rs11662485
rs10058711
rs191852210
All − MR Egger
All − Inverse variance weighted
−0.8
−0.4
0.0
0.4
MR effect size for
'finngen_R9_M13_OSTEOPOROSIS' on 'GCST90274774.tsv.gz'
